# Intraoperative Boost Radiotherapy during Targeted Oncoplastic Breast Surgery: Overview and Single Center Experiences

**DOI:** 10.1155/2014/637898

**Published:** 2014-12-17

**Authors:** Wolfram Malter, Verena Kirn, Lisa Richters, Claudius Fridrich, Birgid Markiefka, Rudolf Bongartz, Robert Semrau, Peter Mallmann, Stefan Kraemer

**Affiliations:** ^1^Breast Center, University Hospital of Cologne, Kerpenerstrasse 34, 50931 Cologne, Germany; ^2^Department of Obstetrics and Gynaecology, University Hospital of Cologne, Kerpenerstrasse 34, 50931 Cologne, Germany; ^3^Department of Pathology, University Hospital of Cologne, Kerpenerstrasse 34, 50931 Cologne, Germany; ^4^Department of Radiotherapy, University Hospital of Cologne, Kerpenerstrasse 34, 50931 Cologne, Germany

## Abstract

Breast-conserving surgery followed by whole-breast irradiation is the standard local therapy for early breast cancer. The international discussion of reduced importance of wider tumor-free resection margins than “tumor not touching ink” leads to the development of five principles in targeted oncoplastic breast surgery. IORT improves local recurrence risk and diminishes toxicity since there is less irradiation of healthy tissue. Intraoperative radiotherapy (IORT) can be delivered in two settings: an IORT boost followed by a conventional regimen of external beam radiotherapy or a single IORT dose. The data from TARGIT-A and ELIOT reinforce the conviction that intraoperative radiotherapy during breast-conserving surgery is a reliable alternative to conventional postoperative fractionated irradiation, but only in a carefully selected population at low risk of local recurrence. We describe our experiences with IORT boost (50 kV energy X-rays; 20 Gy) in combination with targeted oncoplastic breast surgery in a routine clinical setting. Our experiences demonstrate the applicability and reliability of combining IORT boost with targeted oncoplastic breast surgery in breast-conserving therapy of early breast cancer.

## 1. Breast Surgery in an Oncoplastic Approach

As a standard treatment for early breast cancer, modified radical mastectomy has been replaced by breast-conserving surgery (BCS) (partial mastectomy) followed by breast irradiation. In comparison to modified radical mastectomy, the 20-year survival of partial mastectomy with radiation is not statistically different [1–3]. For BCS, we can use different techniques, for example, quadrantectomy (wide excision), segmentectomy (wide local excision), and lumpectomy (local excision). As demonstrated in the Milan-2 trial, the local recurrence rate was lower for quadrantectomy compared to lumpectomy (3,1% versus 8,1%) [4]. The minimization of local recurrence rates is of importance, because local recurrences are associated with reduced survival and emotional distress [5]. Most local recurrences occur at the site of initial tumor excision or in the same breast quadrant. The incidence of local recurrence depends upon the tumor margin status, patients' age, histology subtype, and adjuvant therapy (local and systemic) [6]. One important factor in determining the risk of local recurrence is the molecular intrinsic subtype of the tumor. The highest risk for local recurrences was demonstrated in tumors that are ER-negative, PR-negative, and Her2-negative (triple negative, TNBC). This risk is independent of breast-conserving therapy or mastectomy [7, 8]. Therefore, the triple negative breast cancer subtype is not an indication for mastectomy.

But actually the size of adequate tumor-free resection margin width is discussed controversially [9]. In a meta-analysis of 21 retrospective studies published by Houssami et al., the impact of surgical margins on local recurrence in women was examined in early-stage invasive breast cancer treated with breast-conserving therapy [10]. The diagnosis of positive margins was associated with an odds ratio for local recurrence of 2,42 (*P* < 0.001). No statistical difference was associated with tumor-free resection margins of more than 1 mm after the use of adjuvant systemic and local therapy, considering that “tumor not touching ink” is an adequate local resection margin for BCS. At the moment, the national guidelines require a free margin for invasive cancer of 1 mm and for ductal carcinoma in situ (DCIS) of 2 mm.

The resection of smaller tumor sizes and of unifocal localisations should lead to a favorable cosmetic outcome in the hands of a trained breast surgeon. On the other hand, approximately 10% to 30% of patients are dissatisfied with the aesthetic result after partial mastectomy and adjuvant radiation [11–13]. For these failure many possibilities causes, p.e. tumor resection can produce distortion, retraction, and noticeable volume changes in the breast. Changes in the position of the nipple-areola complex can extenuate asymmetry. Also, the radiotherapy can have a strong influence on the treated breast: edema, skin erythema, hyperpigmentation, fibrosis, and retraction. To avoid these effects, a concept of oncoplastic techniques in breast-conserving surgery was introduced to combine optimized oncological local safety with optimized aesthetic outcomes concerning partial mastectomy defect reconstruction and remodelling of size, contour, and symmetry of the breast [14].

## 2. Principles and Systematics of Oncoplastic Breast Surgery

The interrelationship between breast-tumor ratio, volume loss, cosmetic outcome, and margins of clearance is complex, and the widespread popularity of breast-conserving surgery has focused attention on new oncoplastic techniques that can avoid unacceptable cosmetic results. Until now, surgical options have been limited to breast-conserving surgery or mastectomy, the choice depending on fairly well-defined indications and factors. Oncoplastic procedures provide a third option that avoids the need for mastectomy in selected patients and can influence the outcome of breast-conserving surgery in three ways [14].

Oncoplastic procedures allow wide local excisions of breast tissue without risking major local defects and deformity. The use of oncoplastic techniques to prevent deformity can extend the scope of breast-conserving surgery, without compromising the adequacy of resection or the cosmetic outcome. Volume replacement can be used after previous breast-conserving surgery and radiotherapy to correct unacceptable deformity and may prevent the need for mastectomy in some cases of local recurrence when further local excision will result in considerable volume loss.

The choice of technique depends on a number of factors, including the extent of resection, location of the tumour, timing of surgery, experience of the breast surgeon in oncoplastic techniques, and expectations of the patient [13, 15–18]. Partial mastectomy reconstruction at the same time as resection is gaining popularity. As a general rule, it is much easier to prevent than to correct a deformity, as the sequelae of previous surgery do not have to be addressed [19]. Immediate reconstruction at the time of partial mastectomy is associated with clear surgical, financial, and psychological benefits [20–22].

Resection defects can be reconstructed in one of two ways—(a) by volume displacement with recruiting and transposing local glandular or dermoglandular flaps into the resection site or (b) by volume replacement, importing volume from elsewhere to replace the amount of tissue resected. Volume replacement techniques can restore the shape and size of the breast, achieving symmetry and excellent cosmetic results without the need for contralateral surgery. However, these techniques require additional operation time and may be complicated by donor-site morbidity, flap loss, and an extended reconvalescence. In contrast, volume displacement techniques require less extensive surgery, limiting scars to the breast and avoiding donor-site problems. There are more than 200 different oncoplastic techniques published. We predefined five surgical principles in* targeted oncoplastic breast surgery*, which are learnable and teachable in an academic institution and result in good or excellent cosmetic outcomes within breast-conserving therapy in more than 95% of treated patients. These surgical principles are glandular rotation, dermoglandular rotation, tumor-adapted reduction mammoplasty, combination of BCT and thoracoepigastric flap, or combination of BCT and latissimus dorsi flap to reconstruct hemimastectomy defects. These oncoplastic techniques are published elsewhere in detail [17, 18].

## 3. Intraoperative Radiotherapy (IORT)

Conventional treatment for stages I and II breast cancer consists of breast-conserving therapy with segmentectomy, surgical axillary staging (sentinel lymph node biopsy), and whole-breast radiotherapy. Postoperative adjuvant radiotherapy is generally delivered 6 weeks after surgery in fractionated daily doses during 5-6 weeks and also as a boost over the tumor bed. The EORTC 22881-10882 trial showed that an additional boost of 16 Gy reduces the risk of local recurrence in about 4% in 10.8 years [23]. Approximately 85% of local recurrences appear in tissue adjacent to the primary tumor after conservative surgery within 5 years of follow-up.

In intraoperative radiotherapy (IORT), a single radiation dose is delivered under direct, visual inspection of the tumor bed. It thus improves local recurrence risk and diminishes toxicity since there is less irradiation of healthy tissue. Intraoperative radiotherapy (IORT) can be delivered in two settings: an IORT boost followed by a conventional regimen of external-beam radiotherapy or a single IORT dose [24, 25].

In The Lancet, Vaidya et al. present results of the TARGIT-A trial, while in The Lancet Oncology, Umberto Veronesi and colleagues present results of the ELIOT trial. Each trial compared a different type of single intraoperative radiotherapy with external whole-breast irradiation.

The TARGIT-A findings add to the first report [26]. This noninferiority study compared one intraoperative dose of 20 Gy using a spherical applicator (point source of 50 kV energy X-rays) with whole-breast irradiation. Breast-conserving surgery was performed using* lumpectomy*. In Germany, a tumor-free resection margin of at least 10 mm was recommended to avoid whole-breast irradiation. Overall, the 5-year risks for local recurrence in the conserved breast for intraoperative radiotherapy versus whole-breast irradiation were 3.3 (95% CI 2.1–5.1) versus 1.3 (95% CI 0.7–2.5; *P* = 0.042). These results are acceptable in terms of the threshold of the predefined noninferiority margin of 2.5% [27].

In the ELIOT trial, 1305 patients were randomised after* quadrantectomy* to receive either whole-breast irradiation (50 Gy in 25 fractions followed by a boost of 10 Gy in five fractions using an external electron beam without node irradiation) or single intraoperative radiotherapy with electrons (21 Gy in one fraction to the tumour bed using electrons of 6–9 MeV). Local recurrence of less than 7.5% in the intraoperative radiotherapy group was deemed to show equivalent efficacy compared with whole-breast irradiation. After median follow-up of 5.8 years, the 5-year event rate for IBTR was 4.4% (95 CI 2.7–6.1) with intraoperative radiotherapy and 0.4% (95% CI 0.0–1.0) with whole-breast irradiation. Thus, the rate of local recurrence with intraoperative radiotherapy was within the prespecified equivalence margin but was significantly worse than that for whole-breast irradiation. Occurrence of true local relapses, local relapses outside the index quadrant, and axillary or regional lymph node metastases was significantly increased with single intraoperative radiotherapy. Of 35 local recurrences in the intraoperative radiotherapy group of ELIOT, 14 (40%) occurred outside the index quadrant and 21 (60%) were local recurrences within the index quadrant [28].

To date, there have been only a few publications of studies with short-term follow-up in which IORT, provided as a boost, demonstrated the potential to prevent local recurrences in early breast cancer (2.6% at 5 years) with good to excellent cosmetic results [29]. Additional open questions are the lack of the final histopathologic report when IORT is applied, the uncertainty regarding the definition of the resection margins, and the resected irradiated volume after repeat resection.

## 4. Cologne-Experiences with IORT Boost

To date, in the Breast Center of the University Hospital of Cologne, according to national guidelines IORT is used outside clinical trials only as a boost radiation followed by whole-breast irradiation. A mobile IORT device generating low-energy X-rays (50 kV) has been used since 2010 for intraoperative radiation in nonlobular breast cancer. This IORT system is applicable to all predefined targeted oncoplastic breast-conserving surgery principles [30]. After oncoplastic wide local excision (segmentectomy) of the tumor, the applicator of the mobile device Intrabeam (Carl Zeiss Surgical, Oberkochen, Germany) is placed into the tumor bed [31]. Using purse-string sutures, the segmentally oriented resection margins of the tumor bed are narrowed to the spherical applicator. To prevent skin toxicity, skin margins were everted before starting IORT. Thereafter, a single dose of 20 Gy was provided at the applicator surface. After complete wound healing and/or chemotherapy, whole-breast radiotherapy was initiated. The median treatment time of the boost intraoperatively was 30 minutes. Outpatient treatment is shortened by 1-2 weeks as a result of the omission of the external-beam boost.

**Figure 1:**
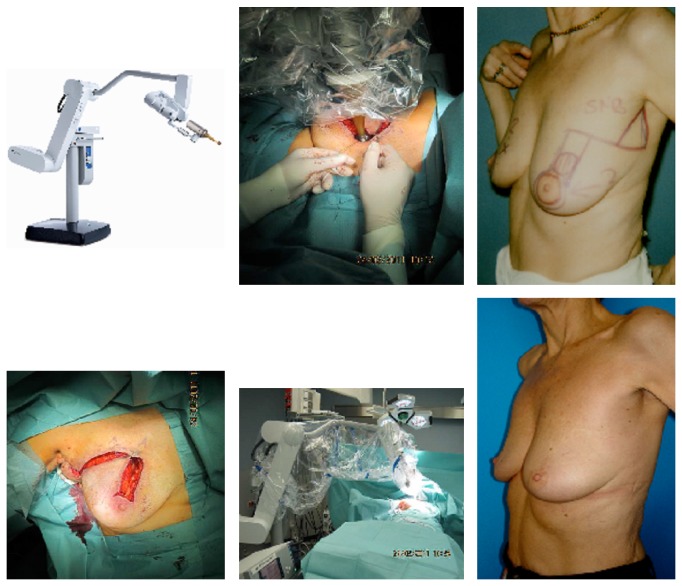
Combination of IORT boost irradiation with targeted oncoplastic breast-conserving surgery (dermoglandular rotation).

Since 2011, a total of 149 patients were treated with IORT as a boost during primary targeted oncoplastic breast-conserving surgery, followed by whole-breast radiotherapy [32]. After mobilisation of glandular tissue, the segmental resection borders were narrowed to the IORT-applicator using purse-string sutures. Resection defects were definitely reconstructed after IORT boost using the predefined oncoplastic principles to achieve optimal esthetic results after breast-conserving surgery (Figure 1). Treated tumors and methodological details of IORT boost are outlined in Table 1. The median age of the patients was 58 (36–86) years. There were T1 and T2 tumors in 117 and 29 patients, respectively, and N0, N1, and N2 diseases in 111, 26, and 12 patients, respectively. The used IORT-applicator sizes ranged between 25 and 40 mm in 79% of the patients. The mean radiation time was 21 (18-32) minutes. IORT boost radiotherapy was combined with oncoplastic principles for partial mastectomy reconstruction as follows: glandular rotation (*n* = 109), dermoglandular rotation (*n* = 29), and tumor-adapted reduction mammoplasty (*n* = 11). Seroma formation 4 weeks after oncoplastic surgery and IORT boost was only observed in 2%. The esthetic outcomes were excellent in more than 90% in patients' view.

## 5. Intraoperative Radiotherapy (IORT) and Targeted Oncoplastic Breast Surgery

To achieve low short-term complication rates (i.e., seroma formation) and good to excellent cosmetic results after breast-conserving surgery and IORT with histologically proven tumor-free resection margins of at least 10 mm in the TARGIT-A trial in Europe, there is a need for oncoplastic breast surgery from a surgical and cosmetic perspective. Despite the new international guidelines in breast-conserving surgery that adequate local surgery is achieved when “tumor is not touching ink,” there is a rationale for a breast-surgery concept we termed targeted oncoplastic breast surgery. Depending on localisation, size of the tumor compared to the size of the breast, and skin involvement, we predefined five principles for partial mastectomy reconstruction [17, 18].

The outcome of early breast cancer is depending on a combination of tumor biology, tumor burden, and adequate adjuvant local and systemic therapy in a multimodality treatment strategy.* From our perspective, to achieve an optimized outcome in early breast cancer, IORT as a boost with additional whole-breast irradiation or as a single dose should be combined with this concept of targeted oncoplastic breast surgery* and evidence-based adjuvant systemic treatment.

## 6. Conclusion

The data from TARGIT-A and ELIOT reinforce the conviction that intraoperative radiotherapy during breast-conserving surgery is a reliable alternative to conventional postoperative fractionated irradiation, but only in a carefully selected population at low risk of local recurrence. Since recurrence can occur after a considerable time delay, final assessment of IORT will only be valid after sufficient follow-up from the prospective randomised trials. Until then, a single IORT dose should be considered experimental. According to national guidelines in our institution, only IORT boost followed by whole-breast irradiation is performed routinely outside a clinical trial. To overcome early and late side-effects and to achieve an optimized aesthetic outcome,* IORT boost* is performed after segmental resection in a concept of* targeted oncoplastic breast surgery* on a routine basis in our institution.

## Figures and Tables

**Table 1:** Treated tumors and methodological details of IORT boost.

	*n*
Patients treated	149
Tumor size	
pT1	117
pT2	29
pT3	3
Nodal status	
pN0	111
pN1	25
pN2	12
Grading	
G1	25
G2	99
G3	29
Tumor biology	
ER +	131
PR +	133
HER2 +	12
Targeted oncoplastic breast surgery principles	
Glandular rotation	109
Dermoglandular rotation	29
Tumor-adapted reduction mammoplasty	11
IORT applicator size	
15 mm	2
20 mm	7
25 mm	29
30 mm	46
35 mm	41
40 mm	20
45 mm	4

## References

[1] Veronesi U., Cascinelli N., Mariani L. (2002). Twenty-year follow-up of a randomized study comparing breast-conserving surgery with radical mastectomy for early breast cancer. *The New England Journal of Medicine*.

[2] Fisher B., Anderson S., Bryant J., Margolese R. G., Deutsch M., Fisher E. R., Jeong J.-H., Wolmark N. (2002). Twenty-year follow-up of a randomized trial comparing total mastectomy, lumpectomy, and lumpectomy plus irradiation for the treatment of invasive breast cancer. *The New England Journal of Medicine*.

[3] van Dongen J. A. (2009). Long term results of a randomized trial. *Journal of the National Cancer Institute*.

[4] Veronesi U., Volterrani F., Luini A. (1990). Quadrantectomy versus lumpectomy for small size breast cancer. *European Journal of Cancer*.

[5] Darby S., McGale P. (2011). Effect of radiotherapy after breast conserving surgery. *The Lancet*.

[6] Hoffty B. G., Fischer D., Rose M., Beinfield M., McKhann C. (1991). Prognostic factors for local recurrence in the conservatively treated breast cancer patient: a cautious interpretation of the data. *Journal of Clinical Oncology*.

[7] Kyndi M., Sørensen F. B., Knudsen H. (2008). Estrogen receptor, progesterone receptor, HER-2, and response to postmastectomy radiotherapy in high-risk breast cancer: the Danish Breast Cancer Cooperative Group. *Journal of Clinical Oncology*.

[8] Nguyen P. L., Taghian A. G., Katz M. S., Niemierko A., Abi Raad R. F., Boon W. L., Bellon J. R., Wong J. S., Smith B. L., Harris J. R. (2008). Breast cancer subtype approximated by estrogen receptor, progesterone receptor, and HER-2 is associated with local and distant recurrence after breast-conserving therapy. *Journal of Clinical Oncology*.

[9] Morrow M., Harris J. R., Schnitt S. J. (2012). Surgical margins in lumpectomy for breast cancer: bigger is not better. *The New England Journal of Medicine*.

[10] Houssami N., MacAskill P., Marinovich M. L., Dixon J. M., Irwig L., Brennan M. E., Solin L. J. (2010). Meta-analysis of the impact of surgical margins on local recurrence in women with early-stage invasive breast cancer treated with breast-conserving therapy. *European Journal of Cancer*.

[11] Clough K. B., Cuminet J., Fitoussi A., Nos C., Mosseri V. (1998). Cosmetic sequelae after conservative treatment for breast cancer: classification and results of surgical correction. *Annals of Plastic Surgery*.

[12] Rezai M., Veronesi U. (2007). Oncoplastic principles in breast surgery. *Breast Care*.

[13] Kroll S. S., Singletary S. E. (1998). Repair of partial mastectomy defects. *Clinics in Plastic Surgery*.

[14] Dough K. B., Kroll S. S., Audretsch W. (1999). An approach to the repair of partial mastectomy defects. *Plastic and Reconstructive Surgery*.

[15] Clough K. B., Lewis J. S., Couturaud B., Fitoussi A., Nos C., Falcou M.-C. (2003). Oncoplastic techniques allow extensive resections for breast-conserving therapy of breast carcinomas. *Annals of Surgery*.

[16] Audretsch W., Rezai M., Kolotas C. (1998). Tumor-specific immediate reconstruction in breast cancer patients. *Seminars in Plastic Surgery*.

[17] Krämer S., Kümmel S., Camara O., Große R., Friedrich M., Blohmer J.-U. (2007). Partial mastectomy reconstruction with local and distant tissue flaps. *Breast Care*.

[18] Kraemer S., Darsow M., Kummel S., Kimmig R., Rezai M. (2008). Breast-conserving treatment of breast cancer. *Reviews in Obstetrics and Gynecology*.

[19] Slavin S. A., Love S. M., Sadowsky N. L., Grisotti A., Veronesi U., Shestak K. C. (1992). Reconstruction of the radiated partial mastectomy defect with autogenous tissues. *Plastic and Reconstructive Surgery*.

[20] Kurtz J. M. (1995). Impact of radiotherapy on breast cosmesis. *Breast*.

[21] Dean C., Chetty U., Forest A. P. M. (1983). Effects of immediate breast reconstruction on psychosocial morbidity after mastectomy. *The Lancet*.

[22] Raja M. A. K., Straker V. F., Rainsbury R. M. (1997). Extending the role of breast-conserving surgery by immediate volume replacement. *British Journal of Surgery*.

[23] Bartelink H., Horiot J.-C., Poortmans P. M., Struikmans H., Van Den Bogaert W., Fourquet A., Jager J. J., Hoogenraad W. J., Oei S. B., Warlam-Rodenhuis C. C., Pierart M., Collette L. (2007). Impact of a higher radiation dose on local control and survival in breast-conserving therapy of early breast cancer: 10-year results of the randomized boost versus no boost EORTC 22881–10882 trial. *Journal of Clinical Oncology*.

[24] Sauer R., Sautter-Bihl M. L., Budach W., Feyer P., Harms W., Souchan R., Wollwiener D., Kreienberg R., Wenz F. (2007). Accelerated partial breast irradiation. *Cancer*.

[25] Blohmer J. U., Kimmig R., Kümmel S., Costa S.-D., Krämer S., Rezai M. (2008). Intraoperative radiotherapy of breast cancer. *Gynäkologisch-geburtshilfliche Rundschau*.

[26] Vaidya J. S., Tobias J. S., Baum M., Keshtgar M., Joseph D., Wenz F., Houghton J., Saunders C., Corica T., D'Souza D., Sainsbury R., Massarut S., Taylor I., Hilaris B. (2004). Intraoperative radiotherapy for breast cancer. *The Lancet Oncology*.

[27] Vaidya J. S., Wenz F., Bulsara M. (2014). Risk-adapted targeted intraoperative radiotherapy versus whole-breast radiotherapy for breast cancer: 5-year results for local control and overall survival from the TARGIT-a randomised trial. *The Lancet*.

[28] Veronesi U., Orecchia R., Maisonneuve P., Viale G., Rotmensz N., Sangalli C., Luini A., Veronesi P., Galimberti V., Zurrida S., Leonardi M. C., Lazzari R., Cattani F., Gentilini O., Intra M., Caldarella P., Ballardini B. (2013). Intraoperative radiotherapy versus external radiotherapy for early breast cancer (ELIOT): a randomised controlled equivalence trial. *The Lancet Oncology*.

[29] Blank E., Kraus-Tiefenbacher U., Welzel G. (2010). Single-center long-term follow-up after intraoperative radiotherapy as a boost during breast-conserving surgery using low-kilovoltage X-rays. *Annals of Surgical Oncology*.

[30] Malter W., Kirn V., Mallmann P., Kraemer S. (2014). Oncoplastic breast reconstruction after IORT. *Translational Cancer Research*.

[31] Kraus-Tiefenbacher U., Scheda A., Steil V., Hermann B., Kehrer T., Bauer L., Melchert F., Wenz F. (2005). Intraoperative radiotherapy (IORT) for breast cancer using the Intrabeam system. *Tumori*.

[32] Malter W., Puppe J., Rogee K., Wuerstlein R., Semrau R., Bongartz R., Markiefka B., Mallmann P., Kraemer S. (2012). Single center experiences with intraoperative radiotherapy as a boost during oncoplastic breast-conserving surgery. *European Journal of Cancer*.

